# Impact of *Glossina pallidipes* salivary gland hypertrophy virus (GpSGHV) on a heterologous tsetse fly host, *Glossina fuscipes fuscipes*

**DOI:** 10.1186/s12866-018-1276-7

**Published:** 2018-11-23

**Authors:** Güler Demirbas-Uzel, Andrew G. Parker, Marc J. B. Vreysen, Robert L. Mach, Jeremy Bouyer, Peter Takac, Adly M. M. Abd-Alla

**Affiliations:** 1 Insect Pest Control Laboratory, Joint FAO/IAEA Division of Nuclear Techniques in Food & Agriculture, International Atomic Energy Agency, Vienna International Centre, P.O. Box 100, 1400 Vienna, Austria; 2 Institute of Chemical, Environmental and Biological Engineering, Research Area Biochemical Technology, Vienna University of Technology, Gumpendorfer Straße 1a, 1060 Vienna, Austria; 3 Section of Molecular and Applied Zoology, Institute of Zoology, Slovak Academy of Sciences, 845 06 Bratislava, SR Slovakia; 4 Scientica, Ltd., Hybešova 33, 831 06 Bratislava, Slovakia

**Keywords:** Glossinidae, Hytrosaviridae, Longevity, Insemination, Mating ability, Flight propensity

## Abstract

**Background:**

Tsetse flies (Diptera: Glossinidae) are the vectors of African trypanosomosis, the causal agent of sleeping sickness in humans and nagana in animals. *Glossina fuscipes fuscipes* is one of the most important tsetse vectors of sleeping sickness, particularly in Central Africa. Due to the development of resistance of the trypanosomes to the commonly used trypanocidal drugs and the lack of effective vaccines, vector control approaches remain the most effective strategies for sustainable management of those diseases. The Sterile Insect Technique (SIT) is an effective, environment-friendly method for the management of tsetse flies in the context of area-wide integrated pest management programs (AW-IPM). This technique relies on the mass-production of the target insect, its sterilization with ionizing radiation and the release of sterile males in the target area where they will mate with wild females and induce sterility in the native population. It has been shown that *Glossina pallidipes* salivary gland hypertrophy virus (GpSGHV) infection causes a decrease in fecundity and fertility hampering the maintenance of colonies of the tsetse fly *G. pallidipes.* This virus has also been detected in different species of tsetse files. In this study, we evaluated the impact of GpSGHV on the performance of a colony of the heterologous host *G. f. fuscipes*, including the flies’ productivity, mortality, survival, flight propensity and mating ability and insemination rates.

**Results:**

Even though GpSGHV infection did not induce SGH symptoms, it significantly reduced all examined parameters, except adult flight propensity and insemination rate.

**Conclusion:**

These results emphasize the important role of GpSGHV management strategy in the maintenance of *G. f. fuscipes* colonies and the urgent need to implement measures to avoid virus infection, to ensure the optimal mass production of this tsetse species for use in AW-IPM programs with an SIT component.

## Background

Tsetse flies (Diptera: Glossinidae) are the only cyclical vectors of the pathogenic African trypanosomes that cause human African trypanosomosis (HAT) or sleeping sickness and African animal trypanosomosis (AAT) or nagana in sub-Saharan Africa [1]. There are 33 species and subspecies of tsetse flies that all belong to the genus *Glossina*, divided into the Morsitans, Fusca, and Palpalis groups [2]. Although all tsetse species can transfer pathogenic trypanosomes, members of the Palpalis and Morsitans groups are the primary trypanosome vectors [3]. For instance, *G. f. fuscipes* is a significant vector of trypanosomes in central Africa [4], particularly in Uganda and Western Kenya [5]. In the absence of effective vaccines and drugs against HAT and AAT [6] vector control represents the most efficient strategy to manage these diseases in mainly rural areas [7, 8]. Currently accepted tsetse control tactics are the sequential aerosol technique [9], stationary bait methods (traps and targets) [10], the live bait technology [11] and the sterile insect technique (SIT). The SIT is based on the mass-rearing and sterilization of males with ionizing radiation (e.g., gamma irradiation), and the sequential release of adequate numbers of sterile male insects in the target area [12]. Mating between sterile males and wild females will result in non-viable embryos, leading to the gradual reduction of the target insect population [13]. The SIT has proven to be a powerful control tactic for use against tsetse flies and other Diptera as part of area-wide integrated pest management (AW-IPM) approaches [14].

The implementation of AW-IPM programs with an SIT component against tsetse flies poses significant challenges with respect to colonization and mass-rearing of the target species. Many factors, such as infections with pathogens when the insects are reared continuously or under suboptimal rearing conditions [15], might lead to failures in establishing and maintaining large tsetse colonies and, as a consequence, fail to produce insects of adequate quality.

Infections of tsetse flies derived from natural populations and laboratory colonies with the pathogenic salivary gland hypertrophy virus (SGHV) [16–18], a member of the *Glossina* Hytrosavirus genus and *Hytrosaviridae* family have been frequently observed [19]. SGHV is a rod-shaped enveloped virus (100 × 700–1000 nm) containing a large double-stranded DNA genome of 190 kb [19]. The virus infection is mostly asymptomatic in tsetse flies, but in some cases it can lead to the development of salivary gland hypertrophy (SGH) symptoms, which has been associated with a reduction in the flies’ productivity and eventually loss of the colony [20–22]. SGH prevalence of this virus in natural tsetse populations vary across tsetse species and their locations, but are usually low (prevalence of 0.3 to 7%) [23] However, under mass-rearing conditions of *Glossina pallidipes*, high prevalence rates have been observed that were associated with the use of the in vitro membrane feeding technique that favors horizontal transmission of the virus. In *G. pallidipes*, a species that is considered an efficient vector of trypanosomes [24], SGH symptoms were associated with abnormalities of the ovaries and testicular degeneration, leading to reduced productivity in both male and female flies [15, 23, 25, 26]. Data available on prevalence rates of the virus in colonies of *G. pallidipes* showed that colony decline and eventual collapse could not be averted when the SGH infection rate in the colony reached 70% (Abd-Alla et al., 2016). To mitigate the negative effects of the virus on colony performance, several virus management strategies were developed that have proven to be effective [27–29].

Although SGH symptoms have been detected in natural populations of other tsetse species such as *Glossina austeni, G. morsitans morsitans, G. nigrofusca nigrofusca* and *G. pallicera pallicera* [16, 30, 31], no SGH symptoms have been observed in *G. f.* fuscipes but asymptomatic infection was detected [32]. However, in laboratory colony, intra-hemocoelic injections of GpSGHV into five heterologous tsetse species (*G. brevipalpis, G. m. morsitans, G. m. centralis, G. f. fuscipes* and *G. p. gambiensis*) showed a significant increase in the titer of viral DNA, demonstrating the ability to replicate in these heterologous species [33].

The Government of Ethiopia has embarked on an AW-IPM program with an SIT component to eradicate a *G. f. fuscipes* population in the Deme river valley of Southern Ethiopia [34–36]. The campaign required the establishment and expansion of a colony of the target species in the mass-rearing facility in Kality on the outskirts of Addis Ababa. The colony was initiated with seed material from a colony maintained at the Slovak Academy of Sciences (SAS), Slovakia. Although colony growth was acceptable in the initial stages subsequent low productivity and high mortality resulted in a drastic reduction in colony size. Similar observations were made at the SAS, where the colony was lost. It is worth noting that more than one tsetse species is being maintained in both facilities, including *G. pallidipes,* a species known to be harbor GpSGHV, a situation that may facilitate the transmission of GpSGHV from one tsetse species to another especially if both species were fed using the same membrane as was the case in the SAS colonies. It should be noted that SGHV was detected by PCR in natural populations of *G. f. fuscipes* with a prevalence of 25–40% [32] and an increase in virus titer in GpSGHV injected flies has recently been demonstrated [33].

This study was undertaken as part of efforts to understand the possible causes of the poor colony performance. In this study, we report on the impact of GpSGHV on the performance of *G. f. fuscipes* flies using standard quality control parameters, such as adult longevity, female productivity and mortality, flight propensity, mating ability, and insemination rate.

## Methods

### Tsetse flies

The *G. f. fuscipes* flies used in this study originated from a colony that was established from wild collected material in the Central African Republic (CAR) and maintained since 1986 at the Insect Pest Control Laboratory (IPCL) of the Joint FAO/IAEA Division of Nuclear Techniques in Food and Agriculture, Seibersdorf, Austria. Experimental flies were fed for 15–20 min, three times per week with defibrinated bovine blood using an artificial (in vitro) membrane feeding system [37]. The adult flies were held in medium size cages (11 cm diameter × 5 cm high) at a ratio of 1:3 male to female under standard tsetse colony rearing conditions (24 ± 0.5 °C and 75 ± 5% relative humidity (RH)) [38]. The SGHV is not detectable in this colony by PCR.

### Preparation of virus inoculum and intra-hemocoelic injection

The GpSGHV inoculum was prepared from intact hypertrophied salivary glands dissected from a 10-day-old male *G. pallidipes* showing overt SGH symptoms [39]. Briefly, the hypertrophied glands were homogenized in phosphate buffered saline (PBS) at a concentration of one pair of glands/ml and the homogenate was centrifuged at 400 x *g* for 2 min at room temperature. The supernatant was transferred to a new sterile tube and used immediately after preparation of the inoculum.

Using a 1 ml Myjector U-40 Insulin type syringe (Teruma, Leuven, Belgium) either 2 μl of filter-sterilized PBS (control) or the virus suspension was injected into the thoracic cavity of prechilled adult flies. For each treatment, newly emerged teneral (male and female) flies were injected and placed into standard holding cages (20 cm diameter × 5 cm high) at the required mating ratio and each experiment was replicated 3 times. Non-injected and PBS-injected flies were used as non-injected controls to evaluate the impact of injection and the virus infection on the flies’ performance.

### Prevalence of GpSGHV infection in *G. f. fuscipes* injected flies

The tsetse genomic DNA was extracted from individual non-injected, PBS- and virus-injected flies using the DNeasy Blood & Tissue kit (QIAGEN Inc., Valencia, CA) following the manufacturer’s instructions. The titer of GpSGHV was determined in *G. f. fuscipes* injected males and females on 0, 9 and 18 days post injection by polymerase chain reaction (PCR) using the method previously described by Abd-Alla et al. [20]. Equal volume of individual DNA sample was pooled (*n* = 6 for females and *n* = 2 for males) and measured to determine the DNA concentration by spectrophotometry (Nanodrop-Synergy H1 Multi-Mode Reader, BioTek, Instruments, Inc., USA), DNA samples were diluted to final concentration of 4 ng/μl and 5 μl was used as template for the qPCR reaction. The qPCR was performed with odv-e66 (GpSGHV ORF5) gene using the method previously described [20, 39, 40] and the tsetse *β-tubulin* gene was used as a housekeeping gene to normalize the qPCR reactions.

### Impact of GpSGHV infection on survival and productivity of *G. f. fuscipes*

To evaluate the impact of GpSGHV challenge on *G. f. fuscipes,* their productivity and longevity under both normal feeding (blood meal offered three times per week) and starvation stress (no blood feeding) conditions was monitored in non-, PBS- and virus-injected flies. For each treatment, seven males and twenty-one females were kept in standard holding cages and each treatment was replicated 3 times. The productivity data is presented as total pupae over the experimental period per initial female (PPIF).

### Impact of GpSGHV infection on the flight propensity of *G. f. fuscipes*

The flight propensity of virus injected flies, non-injected and PBS-injected flies (the latter two as negative controls) was assessed at 7, 14, 21, 28, 35 and 42 days post injection under normal feeding conditions. Flight tests were carried out in netted cubic mating cages (45 × 45 × 45 cm) that contain a black Polyvinyl Chloride (PVC) tube (8.9 cm diameter, 3 mm thick wall, 10 cm high). The PVC tube allowed light entering only from the top and the walls were coated with unscented talcum powder to prevent the flies from walking out the tube [41]. Standard FAO/IAEA/USDA protocols (FAO/IAEA/USDA, 2014 http://www-naweb.iaea.org/nafa/ipc/public/QualityControl.pdf) were used with a few modifications i.e. rather than using pupae, the adult flies were chilled at 4 °C for 5 min prior to the test, to enable the transfer to the tube. For each test, seven chilled males and twenty-one chilled females were put in a plastic Petri dish (90 mm diameter) with the base covered by black porous paper, and the number of flies that had escaped from the tube “flier” was recorded during one hour [42]. The light intensity at the top of the tubes was 500 lx. Six replicates were conducted for each treatment.

### Impact of GpSGHV infection on the mating ability and insemination rate of *G. f. fuscipes*

The mating ability and the insemination rate of untreated (normal colony) *G. f. fuscipes* males of different ages (3-, 6-, 9- and 12-days post emergence) were assessed to determine the optimal mating age [43]. Forty (40) teneral males were released in mating cages mating cages (45 × 45 × 45 cm), followed 15 min later by an equal number of 9 - day old virgin females for mating. Mating events were observed under standard tsetse rearing conditions from 9:30 to 12:30 h to cover the morning mating activity peak [44]. The optimal mating age test was replicated 3 times and mating tests of virus-challenged flies were repeated 9 times. All flies were offered a blood meal 24 h before mating to increase the mating rate, and non-fed flies were removed and replaced. The propensity for mating ratio was calculated according to the proportion of females that mated for each treatment [44]. After determining the optimal male mating age, 6 to 9-day old non-injected, PBS-injected, and virus-injected virgin males (40 males) were tested as described above to determine mating ability and insemination rate of experimental flies.

Mating pairs were transferred to small cages (4 cm diameter × 6 cm high) and kept for 24 h, after which the males were removed and the females dissected under a binocular microscope to determine insemination rate. Mated female flies were dissected in PBS under a binocular microscope and the insemination rate and spermathecal contents were assessed subjectively at × 100 magnification using a Carl Zeiss compound microscope [45]. The spermathecal fill and insemination rate were obtained by assessing the content of the spermathecae pairs. Spermathecal fill was scored to the nearest quarter for each spermathecae separately as empty (0), quarter full (0.25), half-full (0.50), three-quarter-full (0.75) and full (1.0), For the statistical analysis, quarter full (0.25), half-full (0.50), three-quarter-full (0.75) were considered as partial fill. The amount of sperm transferred was then computed as the mean spermathecal filling values of the spermathecae pairs [46].

### Statistical analysis

The significance of the virus injections on the various parameters was assessed by an ANOVA test [47]. Pairwise comparisons between group means (PBS vs. virus injections, non-injected vs. virus injections and non-injected vs. PBS injections effect on flies) was than determined by Tukey’s honestly significant difference (HSD) test. The analyses were performed in R [48, 49] using RStudio version 3.4.1. [50] The data was transformed using the Box-Cox procedure from the packages. ggplot2 [51], lattice v0.20–35 [52] and MASS v7.3. [53].

All survival analyses were performed using Graph Pad Prism version 5.0 for Windows (GraphPad Software, San Diego California, USA; graphpad.com). The effect of the treatments on fly longevity was analysed using a Log-rank (Mantel-Cox) test. Differences between treatments pairs were tested using the Bonferroni method. Mean longevity (or age in days at death) was calculated from the sum of the number of live flies on each day until the death of the last fly, divided by the number of flies in the group at the start of the experiment. The level of significance was 0.05 for all statistical analyses.

## Results

### Detection of GpSGHV infection in injected flies

**Fig. 1:**
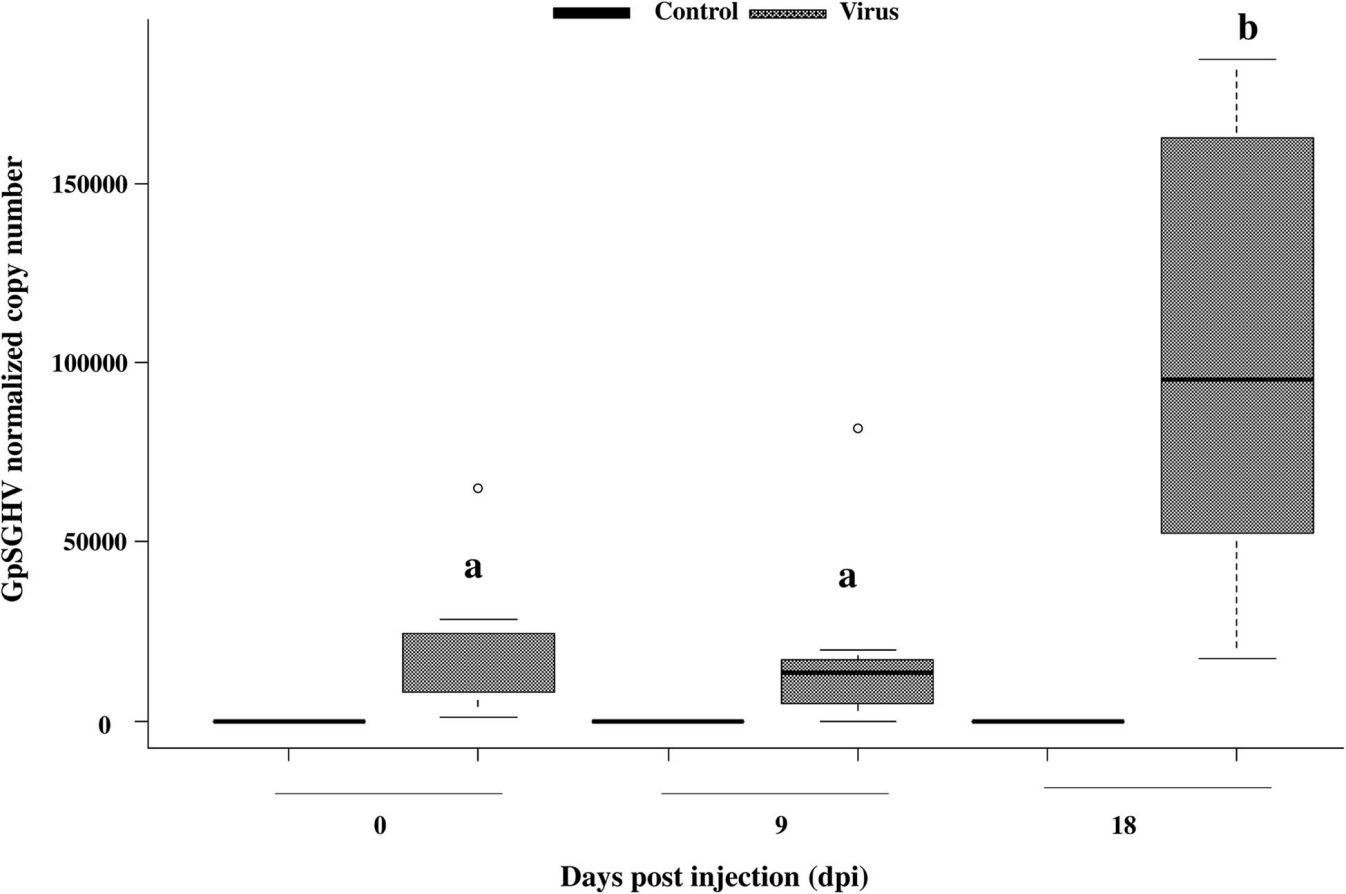
Detection of GpSGHV infection in injected *G. f. fuscipes*. Quantification of GpSGHV titer in virus and PBS- injected flies over 18 day post injection

The GpSGHV titer in virus-injected flies was assessed by qPCR at various times post injection to investigate whether the virus could infect and replicate in injected flies. The qPCR results indicate a significant increase in the virus titer over time (F = 1.34, df = 1, 34, *P* < 0.001). The results indicate that the virus replication was rather slow as no significant increase in the virus titer between 0 time and 9 dpi was observed, but later the virus titer increase by 5. 22 fold change at 18 dpi **(**Fig. 1**)**. In addition, results demonstrated a significant difference in the virus titer between the virus injected flies and negative controls (PBS- injected flies (F = 21.51, df = 1, 68, *P* < 0.001).

### Impact of GpSGHV infection on *G. f. fuscipes* productivity and survival

**Fig. 2:**
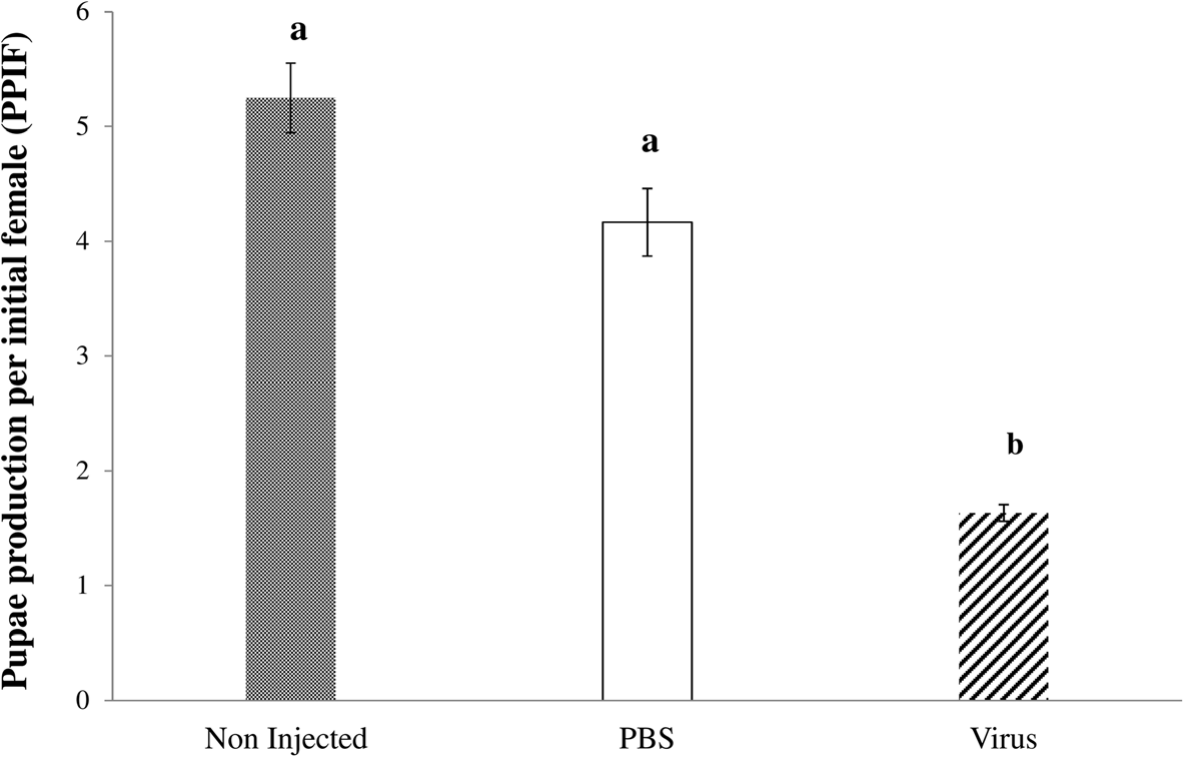
Impact of GpSGHV infection on *G. f. fuscipes* fly productivity and survival. Teneral females were injected with GpSGHV suspension or PBS with non-injected controls. Pupal production per initial female (PPIF) were monitored weekly for 110 days

Virus challenge reduced the productivity of the flies significantly (F = 52.05, df = 2,6, *P* < 0.0001) (Fig. 2). This reduction was significant when compared with PBS-injected (*P* < 0.001) and non-injected (*P* < 0.0005) flies. The injection process had no impact on their productivity as no significant difference (*P* = 0.079) was observed between non-injected and PBS-injected flies.

**Fig. 3:**
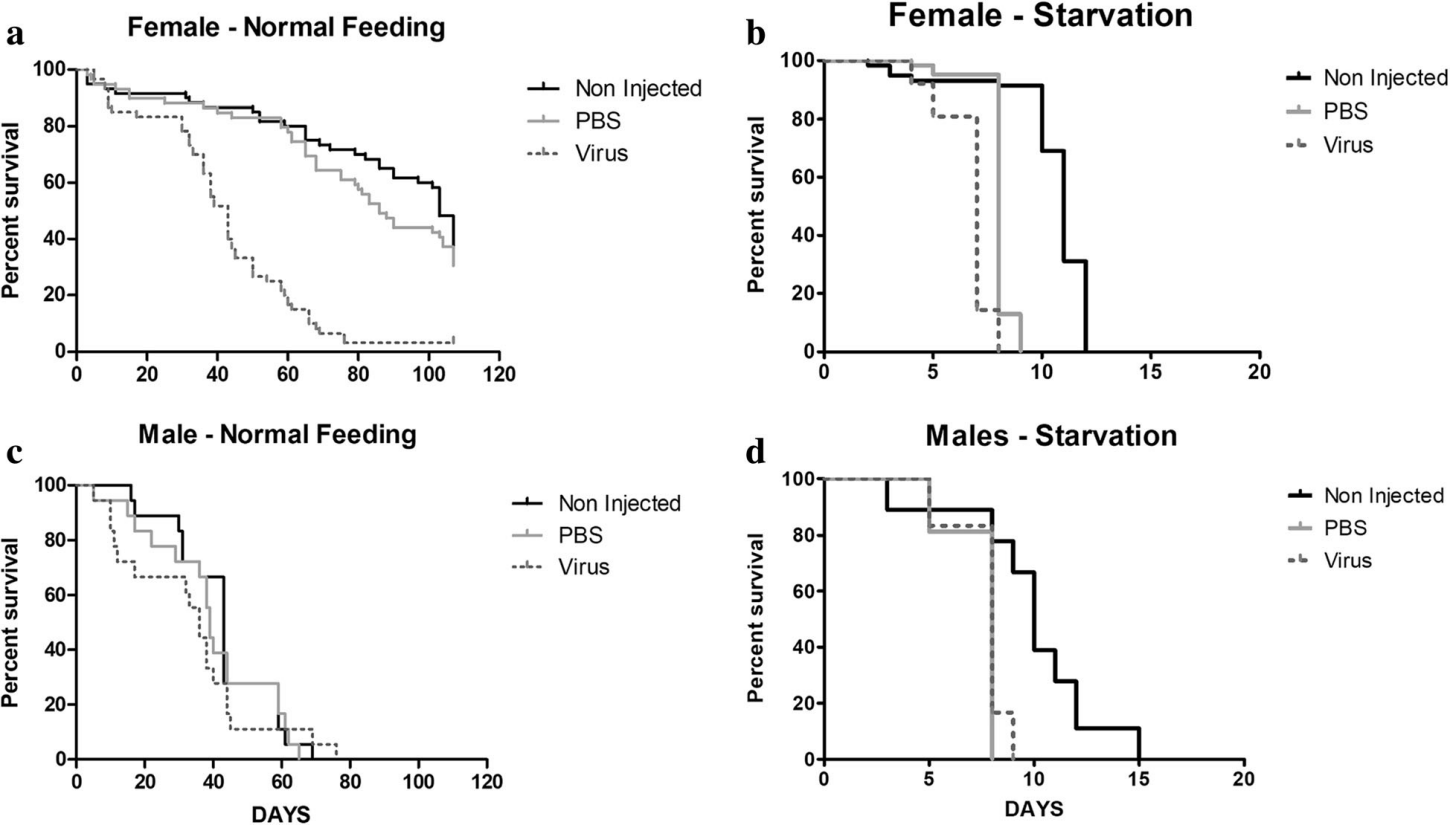
Survival of *G. f. fuscipes* species infected with GpSGHV. **a** and **c**: adult survival under normal feeding condition for females and males respectively. **b** and **d** adult survival under starvation stress conditions for females and males respectively

Adult survival was evaluated under normal feeding and starvation stress conditions. The daily survival rate of the fed virus-injected flies (males and females) was significantly lower than the non-injected and PBS-injected fed flies (Log-rank *X*^2^ = 61.31, df = 2, *P* < 0.0001) (Fig. 3). The mortality rate of the virus-injected flies was higher (100%) than PBS- (75%) and non-injected flies (70%) when measure at 80 days post injection. The survival rate of injected flies varied significantly between males and females (Log-rank X2 = 86.26, df = 3, *P* < 0.0001) **(**Fig. 3**)**. Under normal colony conditions, the survival of GpSGHV-infected females was significantly reduced as compared with PBS-injected females (Log-rank *X*^2^ = 48.3; df = 1, *P* < 0.0001) and non-injected females (Log-rank *X*^2^ = 58.3, df = 1, *P* < 0.0001) (Fig. 3a), however, no significant difference (Log-rank *X*^2^ = 0.50; df = 2, *P* > 0.05) in survival was observed between virus-injected and non-injected males (Fig. 3c).

Under starvation stress, the survival rate of the flies was significantly lower than the survival under normal condition regardless of treatment. However, the virus- and PBS-injected females showed a lower survival (Log-rank *X*^2^ = 87.02, df = 2, *P* < 0.001, less than 10 days) as compared with the non-injected females (Fig. 3b). Similar to female flies, the virus- and PBS-injected males lived a significantly shorter time (Log-rank *X*^2^ = 8.741; df = 2, *P* < 0.001) (less than 10 days, similar to female survival) as compared with the non-injected males (Fig. 3d).

### Flight propensity of GpSGHV injected *G. f. fuscipes*

**Fig. 4:**
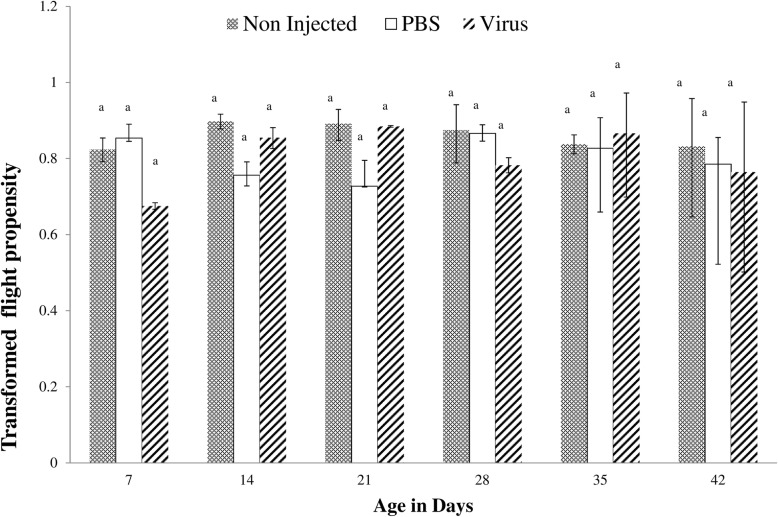
Impact of GpSGHV injection on *G. f. fuscipes* flight propensity at 7, 14, 28, 35 and 42 days post injections (dpi). The data was angular transformed for normality and detransformed for presentation. Mean ± SE

GpSGHV infection had no significant impact (F = 1.4; df = 2, 42; *P* = 0.25) on the flight propensity of *G. f. fuscipes* males and females as compared with the PBS-injected and non-injected flies (Fig. 4). The average percentage of fliers for different treatments was evaluated at different times post emergence (7, 14, 21, 28, 25, 42 days). No significant difference in flight propensity was recorded at different times regardless of treatment (F = 0.08; df = 1, 52; *P* = 0.91).

### Impact of GpSGHV infection on *G. f. fuscipes* flies mating ability

**Fig. 5:**
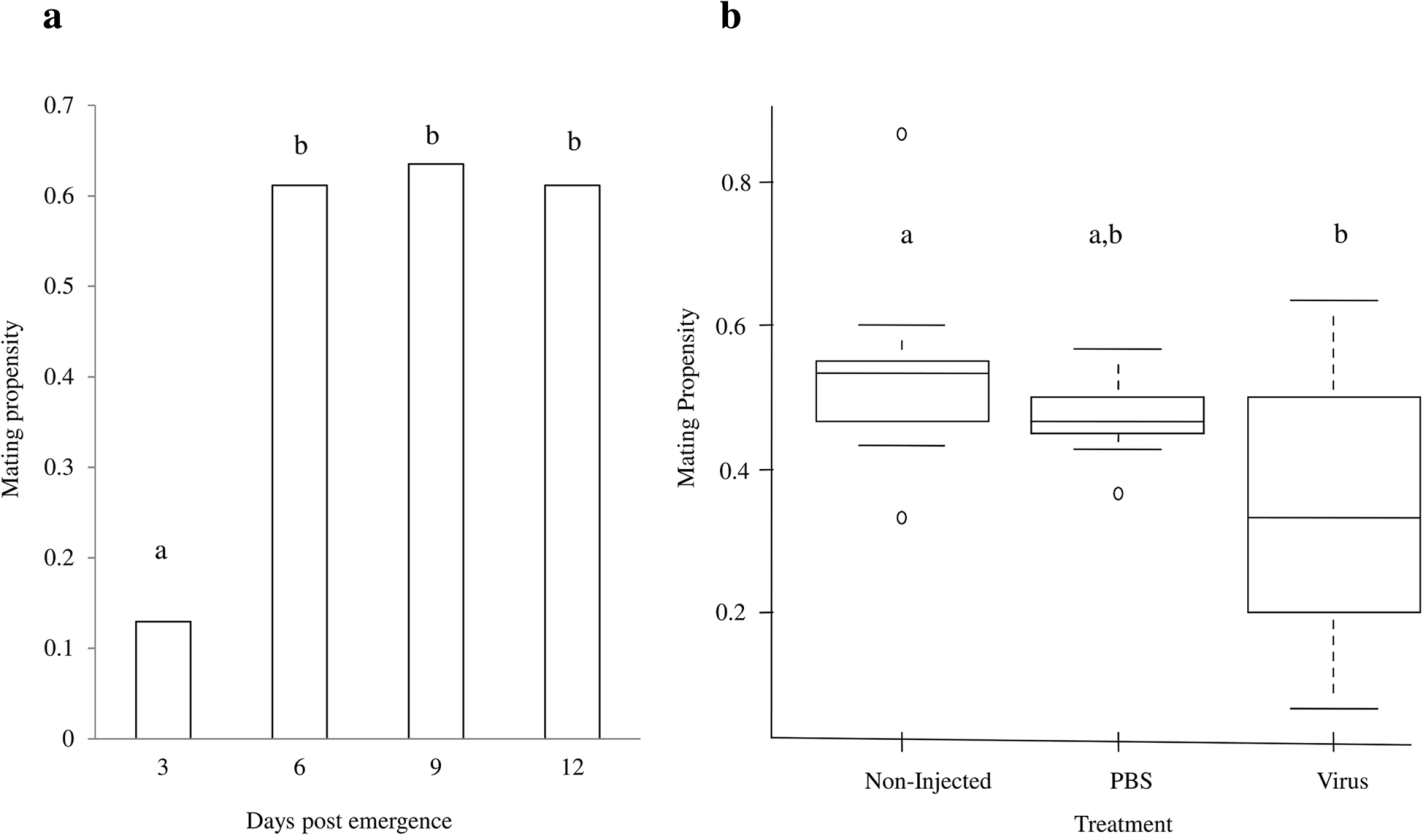
Impact of GpSGHV injection on *G. f. fuscipes* mating ability. **a:** mating propensity of 3, 6, 9 and 12 day old untreated *G. f. fuscipes* males; **b:** Nine day old virgin males from different treatments mated with 9 day old females

In order to assess the impact of the GpSGHV infection of the flies’ mating ability, it was essential to determine the optimal mating age of untreated flies. Mating propensity of 3, 6, 9 and 12 day-old males differed significantly (F = 3.07, df = 3, 8, *P* < 0.001) with 3 day-old males having a significantly lower mating success as compared with older males (*P* < 0.001). However, no significant difference in the mating propensity of 6, 9 and 12 day-old males (*P* > 0.05) was observed **(**Fig. 5a**)**.

Therefore, 6–9 day-old males were used to assess the impact of GpSGHV infection on the mating ability of male flies. Most of the mating pairs were formed in the first hour after introduction of the females into the mating cages, and mating gradually reduced during the remaining 2 h of the test. In general, mating propensity of non-, PBS- and virus-injection flies was significantly different (F = 4.89, df = 2, 24, *P* = 0.016). The mating propensity of virus-injected males was significantly reduced as compared with non-injected males (*P* = 0.014) (Fig. 5b), while, no significant difference was observed between PBS-injected and non-injected males (*P* = 0. 59) or between the PBS and virus-injected males (*P* = 0.11).

### Impact of GpSGHV infection on insemination rate

**Fig. 6:**
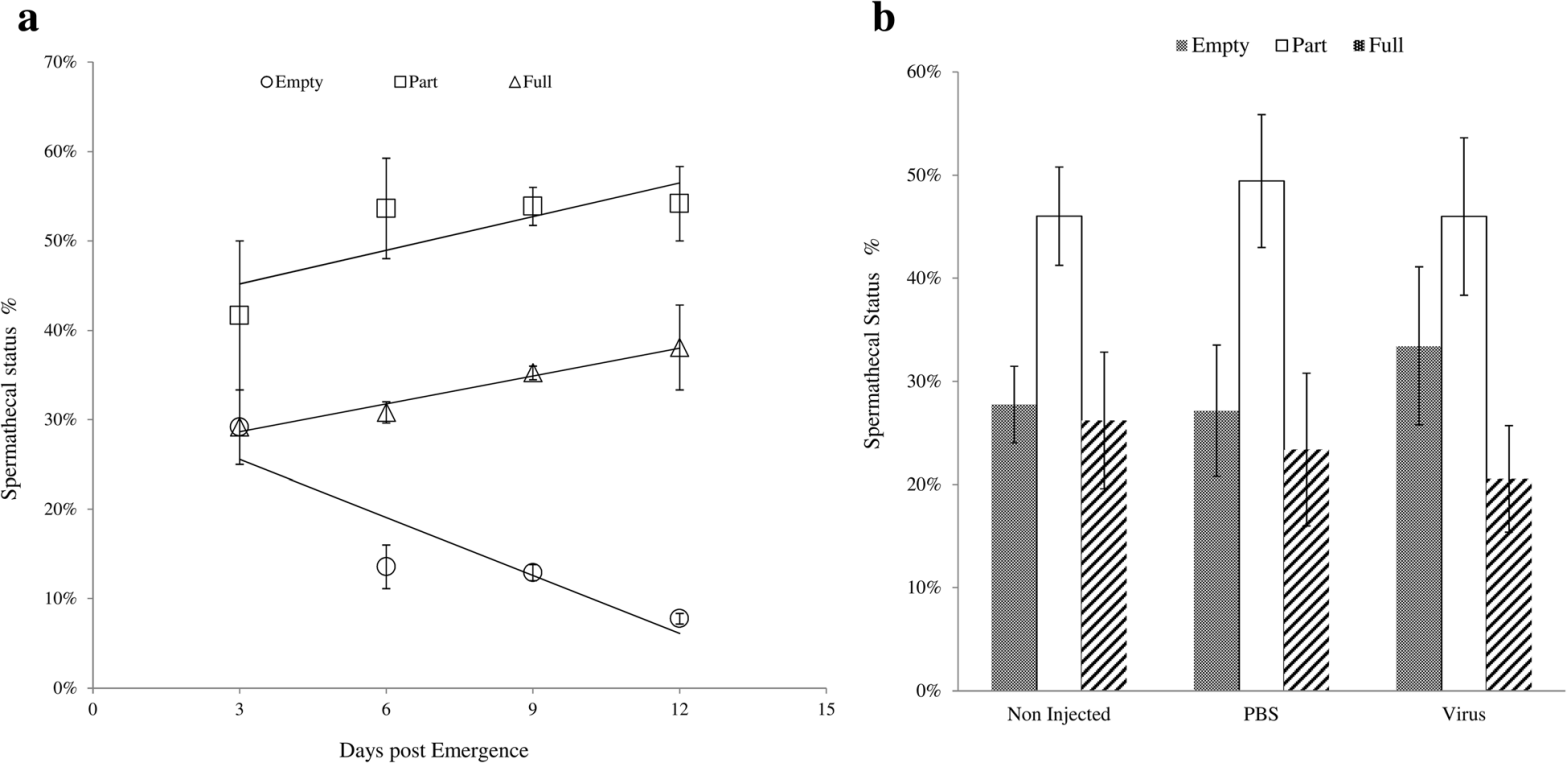
Impact of GpSGHV infection on *G. f. fuscipes* female insemination rate when mated with **a:** 3, 6, 9 or 12 day old untreated males; Proportion of empty spermathecae reduced significantly with male age (y = − 0.02165*x + 0.3207, *P* = 0.005); proportion partially filled (y = − 0.01257*x + 0.4139, *P* = 0.1577) and completely filled increased (y = 0.01040 * x + 0.25526, *P* = 0.04 between 3 and 12 days post emergence. **b:** 9-days old virgin males from different treatments

Females mated with untreated males of different ages showed variable insemination rates (Fig. 6a). The proportion of females with empty spermathecae decreased as male age increased (F = 17.89, df = 1, 6, *P* = 0.005). The percentage of females with partially and fully filled spermathecae increased slightly but not significantly (F = 2.6, df = 1, 6*, P* = 0.15) while the percentage of females with fully filled spermathecae increased significantly (F = 6. 74, df. = 1, 6; *P* = 0.04) with increasing age of the males (Fig. 6a).

The GpSGHV injection of the males did not affect the insemination rate for empty, partially and fully filled spermathecae values or empty spermathecal values (F = 0.19, df = 2, 24, *P* = 0.8261) (Fig. 6b).

## Discussion

The challenge in establishing large colonies of tsetse flies in mass-rearing facilities for the implementation of the SIT component in AW-IPM programs has always been a strong driver to explore and identify the key factor(s) affecting tsetse biology. The collapse of colonies of *G. pallidipes* at the IPCL and in Ethiopia prompted a decade of research on the productivity problems in these colonies and its association with the GpSGHV. As a result, virus management strategies have been developed to mitigate the instability in production of these colonies [20, 27–29]. In view of the similarity of low productivity of the *G. pallidipes* and *G. f. fuscipes* colonies maintained at the SAS in Bratislava, Slovakia and Addis Ababa, Ethiopia, this study was conducted to investigate whether a potential GpSGHV infection might contribute to the low performance of the *G. f. fuscipes* colony. Our data indicates that the presence of the virus indeed reduced various important quality parameters such as adult longevity, female productivity and male mating ability and, in addition, increased the mortality rate. Conversely, flight ability and insemination rate of virus-challenged flies was not affected as compared with uninfected ones.

Despite the negative impact of virus challenge on the flies’ performance, no SGH symptoms were observed in injected *G. f. fuscipes* flies and no virus transmission to the F_1_ progeny was detected (data not shown). These findings are in agreement with recent data demonstrating that the GpSGHV can replicate in five heterologous tsetse species without inducing SGH or being vertically transmitted to the F_1_ offspring [33]. The results also agree with previously reported data on the significant reduction in the lifespan of *G. pallidipes* challenged with GpSGHV [54]. In addition, similar results were obtained by injecting the house fly *Musca domestica* salivary gland hypertrophy virus in a heterologous host, *Stomoxys calcitrans*, where the infection had a negative impact on survival and fecundity of the heterologous host without the development of SGH symptoms [55].

The GpSGHV infection in *G. f. fuscipes* affected fitness parameters such as increased mortality and reduced fecundity which are the key parameters for colony stability and growth. Moreover, the effects of virus infection impacted females more than males. This is especially relevant for tsetse flies whose fecundity is low [56]. This negative impact on female mortality and productivity under normal colony conditions may explain the problems in maintaining the colonies (both in Slovakia and in Ethiopia) and its ultimate reduction in colony numbers. The lower female survival due to the presence of the virus agrees with previous reports on *G. pallidipes*, showing that females with apparent viral infection as indicated by their enlarged salivary glands had a significantly shorter lifespan than females with normal salivary glands [57]. Likewise, the longevity of virus-infected *G. m.centralis* flies was significantly reduced as compared with uninfected control flies [26].

Our observed positive correlation between male age and mating success was in agreement with previously reported that in field cages, males younger than 8 days showed a significant lower mating ability [43]. Our results showed that 3 day-old males were less successful in mating than older males, but no further significant difference was observed between 6-day old or older males. Similar observations were reported with other species, i.e. 3-day old male *G. brevipalpis* and *G. austeni* were less successful in mating as compared with older males [58]. In other studies, 6–8 day old-males *G. p. gambiensis* were used for mating studies [59] and older *G. pallidipes* males copulated more often than young males [44, 60].

The significant reduction of the mating ability of GpSGHV-challenged male *G. f. fuscipes* flies is an additional negative impact of the presence of the virus. The observed reduction in mating success as measured in small mating cages that mimic well the situation in standard tsetse holding cages, might partly explain the reduction in the females’ fecundity as almost half of the females were not inseminated when offered a mating opportunity with virus-injected males. These results are in agreement with previous studies on the mating performance of *G. pallidipes* in small laboratory cages [57] or in walk-in field cages [61]. Our data are also in agreement with results of *Helicoverpa zea* males infected with the Hz-V2 virus, that were slower in approaching healthy females for mating as compared with non-infected males [62, 63]. This reduction in mating propensity might be a result of reduced flying and searching activity for females or possibly a negative selection by females against infected males [64].

Our data to imply a different outcome when compared with the results of Odindo [65] who reported no significant difference in mating performance between symptomatically infected and asymptomatic *G. pallidipes* flies. In addition, in contrast to our study, Jura and Davies-Cole (1992) speculated that SGHV-infected, and hence sterile, *G. pallidipes* males showed increased mating competitiveness and concluded that these males could be used for SIT applications [66]. Although our and the experiments of Odindo [65] and Jura and Davies-Cole [66] were conducted in similar settings (small laboratory cage), the different results are most likely due to the different tsetse species (*G. pallidipes* versus *G. f. fuscipes*) populations or strains used in the study. However, no data are so far available on the impact of the virus in males on the potential selection of females for mating partners. Further studies on the presence of the virus and its impact on the biological mechanisms of mating are necessary.

The virus injection has no significant impact on flight propensity and insemination rate of infected flies. The absence of a negative impact on the adult flight propensity (males and females) observed in this study contradicts the finding of Odindo [64] who speculated that the presence of the virus resulted in reduced physical male activity in *G. pallidipes*. It also contradicts the observation of Burand and Tan [63] who observed that the Hz-1 virus makes the *H. zea* male lazier and slower to move. The reduction in the mating propensity of virus-infected males might be due to reduced physical male activity. This might indicate that the physical activity required for the flight propensity test is much less than that required for successful mating and therefore the infected males had the propensity to fly but lost the ability to conduct normal mating activity.

The absence of any significant impact of the virus infection on insemination rate might be due to the completion of sperm development during the pupal stage in *Glossina* species prior to the virus challenge of the adult stage. The results contradict earlier data indicating that virus infected *G. pallidipes* males with SGH were unable to successfully inseminate females after mating [21, 61]. The difference between our current data and these published earlier might be due to a different level of virus infection (virus infected *G. f. fuscipes* showed no sign of SGH versus *G. pallidipes* males with SGH indicating a higher density of virus particle per flies (> 10^6^) and a different tsetse species.

## Conclusions

The data presented in this paper directly demonstrates the negative impact of GpSGHV infection on the establishment and maintenance of *G. f. fuscipes* colonies, which will be crucial for the production of sufficient male flies of adequate biological quality for the application of the SIT programmes. The combination of increased female fly mortality and the reduction in mating propensity of the virus-infected males will shorten the production period and therefore will necessitate an increase in colony size to compensate for the loss in production. Finally, virus-infected males might have a lower competitiveness under field conditions, which will require increased release rates. These combined effects of the presence of the virus in *G. f. fuscipes* colonies will impose serious challenges to mass-rear and produce sufficient sterile males of adequate biological quality and will make the SIT component more expensive and less competitive with other control tactics [67]. Management strategies to mitigate the negative effects of virus presence that were based on the use of a clean feeding system (each fly receives a clean blood meal) and the mixing of the blood meals with the antiviral drug valacyclovir were recently developed for *G. pallidipes* colonies. However, the implementation of these strategies has so far been restricted to *G. pallidipes* colonies where flies showed clear SGH symptoms [27–29]. So far, the absence of SGH symptoms in many tsetse species including *G. f. fuscipes* has excluded the virus-infection as a possible cause for the poor performance of certain colonies and consequently no virus management strategies were implemented. The data presented in this manuscript strongly indicates that colonies that perform poorly should be screened for the presence of the virus with PCR and in confirmed cases, virus management strategies should be implemented even when no SGH symptoms are observed. Special caution is required in those tsetse mass-rearing facilities where *G. pallidipes* colonies are maintained with colonies of other tsetse species.

## Abbreviations

AAT: animal African trypanosomosis
AW-IPC: area-wide integrated pest management programs
GpSGHV: *Glossina pallidipes* salivary gland hypertrophy virus
HAT: Human African trypanosomosis
IPCL: Insect Pest Control Laboratory
PBS: Phosphate Buffer Saline
qPCR: quantitative polymerase chain reaction
SAS: Slovak Academy of Sciences
SGHV: salivary gland hypertrophy virus
SIT: Sterile Insect Technique

## References

[1] Hotez PJ, Kamath A (2009). Neglected tropical diseases in sub-saharan Africa: review of their prevalence, distribution, and disease burden. PLoS Negl Trop Dis.

[2] Gooding RH, Feldmann U, Robinson AS, Crampton JM, Beard CB, Louis C (1997). Care and maintenance of tsetse colonies. The molecular biology of insect disease vectors: a methods manual.

[3] Aksoy S, Maudlin I, Dale C, Robinson AS, O'Neill SL (2001). Prospects for control of African trypanosomiasis by tsetse vector manipulation. Trends Parasitol.

[4] Rogers DJ, Robinson TP (2004). Tsetse distribution. The trypanosomiases.

[5] Waiswa C, Picozzi K, Katunguka-Rwakishaya E, Olaho-Mukani W, Musoke RA, Welburn SC (2006). *Glossina fuscipes fuscipes* in the trypanosomiasis endemic areas of south eastern Uganda: apparent density, trypanosome infection rates and host feeding preferences. Acta Trop.

[6] Aksoy S, Gibson WC, Lehane MJ (2003). Interactions between tsetse and trypanosomes with implications for the control of trypanosomiasis. Adv Parasitol.

[7] Tirados I, Esterhuizen J, Kovacic V, Mangwiro TC, Vale GA, Hastings I, Solano P, Lehane MJ, Torr SJ (2015). Tsetse control and Gambian sleeping sickness; implications for control strategy. PLoS Negl Trop Dis.

[8] Abd-Alla AMM, Bergoin M, Parker A, Maniania NK, Vlak JM, Bourtzis K, Boucias DG, Aksoy S (2013). Improving sterile insect technique (SIT) for tsetse flies through research on their symbionts and pathogens. J Invertebr Pathol.

[9] Jordan AM (1974). Recent developments in the ecology and methods of control of tsetse flies (*Glossina* spp.) (Dipt., Glossinidae) - a review. Bull Entomol Res.

[10] Green CH (1994). Bait methods for tsetse fly control. Adv Parasitol.

[11] Thompson JW, Mitchell M, Rees RB, Shereni W, Schoenfeld AH, Wilson A (1991). Studies on the efficacy of deltamethrin applied to cattle for the control of tsetse flies (*Glossina* spp.) in southern Africa. Trop Anim Health Prod.

[12] Vreysen MJB (2001). Principles of area-wide integrated tsetse fly control using the sterile insect technique. Med Trop.

[13] Knipling EF (1963). Potential role of the sterility principle for tsetse eradication. WHO/Vector Control/27.

[14] Hendrichs JP, Kenmore P, Robinson AS, Vreysen MJB, Vreysen MJB, Robinson AS, Dordrecht HJ (2007). Area-wide integrated pest management (AW-IPM): principles, practice and prospects. Area-wide control of insect pests: from research to field implementation.

[15] Abd-Alla AMM, Parker AG, Vreysen MJB, Bergoin M (2011). Tsetse salivary gland hypertrophy virus: Hope or hindrance for tsetse control?. PLoS Negl Trop Dis.

[16] Gouteux J-P (1987). Prevalence of enlarged salivary glands in *Glossina palpalis*, *G pallicera*, and *G nigrofusca* (Diptera: Glossinidae) from the Vavoua area, Ivory Coast. J Med Entomol.

[17] Jaenson TG (1978). Virus-like rods associated with salivary gland hyperplasia in tsetse, Glossina pallidipes. Trans R Soc Trop Med Hyg.

[18] Sang RC, Jura WGZO, Otieno LH, Mwangi RW, Ogaja P (1999). The effects of a tsetse DNA virus infection on the functions of the male accessory reproductive gland in the host fly *Glossina morsitans centralis* (Diptera; Glossinidae). Curr Microbiol.

[19] Abd-Alla AMM, Vlak JM, Bergoin M, Maruniak JE, Parker AG, Burand JP, Jehle JA, Boucias DG (2009). Hytrosaviridae: a proposal for classification and nomenclature of a new insect virus family. Arch Virol.

[20] Abd-Alla A, Bossin H, Cousserans F, Parker A, Bergoin M, Robinson A (2007). Development of a non-destructive PCR method for detection of the salivary gland hypertrophy virus (SGHV) in tsetse flies. J Virol Methods.

[21] Abd-Alla AMM, Kariithi HM, Parker AG, Robinson AS, Kiflom M, Bergoin M, Vreysen MJB (2010). Dynamics of the salivary gland hypertrophy virus in laboratory colonies of *Glossina pallidipes* (Diptera: Glossinidae). Virus Res.

[22] Kariithi HM, Ahmadi M, Parker AG, Franz G, Ros VID, Haq I, Elashry AM, Vlak JM, Bergoin M, Vreysen MJB, Abd-Alla AMM (2013). Prevalence and genetic variation of salivary gland hypertrophy virus in wild populations of the tsetse fly *Glossina pallidipes* from southern and eastern Africa. J Invertebr Pathol.

[23] Lietze VU, Abd-Alla AMM, Vreysen MJB, Geden CJ, Boucias DG (2010). Salivary gland hypertrophy viruses: a novel group of insect pathogenic viruses. Annu Rev Entomol.

[24] Moloo SK, Zweygarth E, Sabwa CL (1992). Virulence of *Trypanosoma simiae* in pigs infected by *Glossina brevipalpis, G. pallidipes* or *G. morsitans centralis*. Ann Trop Med Parasitol.

[25] Jura WGZO, Odhiambo TR, Otieno LH, Tabu NO (1988). Gonadal lesions in virus-infected male and female tsetse, *Glossina pallidipes* (Diptera: Glossinidae). J Invertebr Pathol.

[26] Sang RC, Jura WGZO, Otieno LH, Tukei PM, Mwangi RW (1997). Effects of tsetse DNA virus infection on the survival of a host fly *Glossina morsitans centralis* (Diptera: Glossinidae). J Invertebr Pathol.

[27] Abd-Alla AMM, Adun H, Parker AG, Vreysen MJB, Bergoin M (2012). The antiviral drug valacyclovir successfully suppresses salivary gland hypertrophy virus (SGHV) in laboratory colonies of *Glossina pallidipes*. PLoS One.

[28] Abd-Alla AMM, Kariithi HM, Mohamed AH, Lapiz E, Parker AG, Vreysen MJB (2013). Managing hytrosavirus infections in *Glossina pallidipes* colonies: feeding regime affects the prevalence of salivary gland hypertrophy syndrome. PLoS One.

[29] Abd-Alla AMM, Marin C, Parker A, Vreysen M (2014). Antiviral drug valacyclovir treatment combined with a clean feeding system enhances the suppression of salivary gland hypertrophy in laboratory colonies of *Glossina pallidipes*. Parasit Vectors.

[30] Burtt E (1945). Hypertrophied salivary glands in *Glossina*: evidence that *G. pallidipes* with this abnormality is particularly suited to trypanosome infection. Ann Trop Med Parasitol.

[31] Ellis DS, Maudlin I (1987). Salivary gland hyperplasia in wild caught tsetse from Zimbabwe. Entomologia Experimentalis et Applicata.

[32] Meki IK, Kariithi HM, Ahmadi M, Parker AG, Vreysen MJB, Vlak JM, van Oers MM, Abd-Alla AMM: Hytrosavirus genetic diversity and eco-regional spread in *Glossina* species. BMC Microbiol. 10.1186/s12866-018-1297-2. 10.1186/s12866-018-1297-2 PMC6251127 30470191

[33] Demirbas-Uzel G, Kariithi HM, Parker AG, Vreysen MJB, Mach RL, Abd-Alla AMM. Susceptibility of Tsetse Species to *Glossina pallidipes* Salivary Gland Hypertrophy Virus (GpSGHV). Front Microbiol. 2018;9. 10.3389/fmicb.2018.00701. 10.3389/fmicb.2018.00701 PMC5901070 29686664

[34] Alemu T, Kapitano B, Mekonnen S, Aboset G, Kiflom M, Bancha B, Woldeyes G, Bekele K, Feldmann U, Vreysen MJB, Robinson AS, Dordrecht HJ (2007). Area-wide control of tsetse and trypanosomosis: Ethiopian experience in the southern Rift Valley. Area-wide control of insect pests: from research to field implementation.

[35] Feldmann U, Dyck VA, Mattioli RC, Jannin J, Dyck VA, Hendrichs J, Dordrecht RAS (2005). Potential impact of tsetse fly control involving the sterile insect technique. Sterile insect technique. Principles and practice in area-wide integrated Pest management.

[36] Vreysen MJB, Mebrate A, Menjeta M, Bancha B, Woldeyes G, Musie K, Bekele K, Aboset G (1999). The distribution and relative abundance of tsetse flies in the southern Rift Valley of Ethiopia: preliminary survey results. Proceedings of the 25th meeting of the international scientific Council for Trypanosomiasis Research and Control, Mombasa, Kenya, 27 September - 1 October 1999.

[37] Bauer B, Wetzel H (1976). A new membrane for feeding *Glossina morsitans* Westw. (Diptera: Glossinidae). Bull Entomol Res.

[38] Feldmann U, JPR O’-O (1994). Guidelines for the rearing of tsetse flies using the membrane feeding technique. Techniques of insect rearing for the development of integrated pest and vector management strategies.

[39] Boucias DG, Kariithi HM, Bourtzis K, Schneider DI, Kelley K, Miller WJ, Parker AG, Abd-Alla AMM (2013). Transgenerational transmission of the *Glossina pallidipes* hytrosavirus depends on the presence of a functional symbiome. PLoS One.

[40] Abd-Alla AMM, Cousserans F, Parker A, Bergoin M, Chiraz J, Robinson A (2009). Quantitative PCR analysis of the salivary gland hypertrophy virus (GpSGHV) in a laboratory colony of *Glossina pallidipes*. Virus Res.

[41] Pagabeleguem S, Seck MT, Sall B, Vreysen MJB, Gimonneau G, Fall AG, Bassene M, Sidibé I, Rayaissé JB, Belem AMG, Bouyer J (2015). Long distance transport of irradiated male *Glossina palpalis gambiensis* pupae and its impact on sterile male yield. Parasit Vectors.

[42] Hooper GHS (1987). Application of quality control procedures to large scale rearing of the Mediterranean fruit fly. Entomol Exp Appl.

[43] Abila PP, Kiendrebeogo M, Mutika GN, Parker AG, Robinson AS (2003). The effect of age on the mating competitiveness of male *Glossina fuscipes fuscipes* and *G palpalis palpalis*. J Insect Sci.

[44] Mutika GN, Opiyo E, Robinson AS (2001). Assessing mating performance of male *Glossina pallidipes* (Diptera: Glossinidae) using a walk-in field cage. Bull Entomol Res.

[45] Pollock JN (1982). Training manual for tsetse control personnel. Vol.1: tsetse biology, systematics and distribution, techniques.

[46] Nash TAM (1955). The fertilisation of *Glossina palpalis* in captivity. Bull Entomol Res.

[47] Sokal RR, Rohlf FJ (1995). Biometry: the principles and practice of statistics in biological research.

[48] Core Team R (2017). R Core team (2017). R: a language and environment for statistical computing.

[49] Development Core Team (2008). R: a language and environment for statistical computing.

[50] RStudio (2012). RStudio: Integrated development environment for R (Version 0.96.122) [Computer software].

[51] Wickham H (2009). ggplot2: elegant graphics for data analysis.

[52] Sarkar D (2008). Lattice: multivariate data visualization with R.

[53] Venables WN, Ripley BD (2002). Modern applied statistics with S.

[54] Jaenson TGT (1986). Sex ratio distortion and reduced lifespan of *Glossina pallidipes* infected with the virus causing salivary gland hyperplasia. Entomol Exp Appl.

[55] Geden C, Garcia-Maruniak A, Lietze VU, Maruniak J, Boucias DG (2011). Impact of house fly salivary gland hyperthrophy virus (MdSGHV) on a heterologous host, *Stomoxys calcitrans*. J Med Entomol.

[56] Leak SGA (1998). Tsetse biology and ecology: their role in the epidemiology and control of trypanosomosis.

[57] Jaenson TGT (1978). Mating behaviour of *Glossina pallidipes* Austen (Diptera, Glossinidae): genetic differences in copulation time between allopatric populations. Entomol Exp Appl.

[58] de Beer CJ, Venter GJ, Vreysen MJB (2015). Determination of the optimal mating age of colonised *Glossina brevipalpis* and *Glossina austeni* using walk-in field cages in South Africa. Parasit Vectors.

[59] Mutika GN, Kabore I, Seck MT, Sall B, Bouyer J, Parker AG, Vreysen MJB (2013). Mating performance of *Glossina palpalis gambiensis* strains from Burkina Faso, Mali, and Senegal. Entomol Exp Appl.

[60] Alphey L, Andreasen M (2002). Dominant lethality and insect population control. Mol Biochem Parasitol.

[61] Mutika GN, Marin C, Parker AG, Vreysen MJB, Boucias DG, Abd-Alla AMM (2012). Impact of salivary gland hypertrophy virus infection on the mating success of male *Glossina pallidipes*: consequences for the sterile insect technique. PLoS One.

[62] Burand JP, Tan W, Kim W, Nojima S, Roelofs W (2005). Infection with the insect virus Hz-2v alters mating behavior and pheromone production in female *Helicoverpa zea* moths. J Insect Sci.

[63] Burand JP, Tan W (2006). Male preference and mating behaviour of male *Helicoverpa zea* (Lepidoptera: Noctuidae) infected with the sexually transmitted insect virus Hz-2V. Ann Entomol Soc Am.

[64] Odindo MO (1982). Incidence of salivary gland hypertrophy in field populations of the tsetse *Glossina pallidipe*s on the South Kenya coast. Insect Sci Appl.

[65] Odindo MO (1988). *Glossina pallidipes* virus: its potential for use in biological control of tsetse. Insect Sci Appl.

[66] Jura WGZO, Davies-Cole JOA (1992). Some aspects of mating behavior of *Glossina morsitans morsitans* males infected with a DNA virus. Biol Control.

[67] Hendrichs JP, Vreysen MJB, Enkerlin WR, Cayol JP. Strategic options in using sterile insects for area-wide integrated pest management. In: Dyck VA, Hendrichs J, Robinson AS, editors. Dordrecht: Springer; 2005. p. 563–600.

